# QTL Mapping and Validation for Kernel Area and Circumference in Common Wheat *via* High-Density SNP-Based Genotyping

**DOI:** 10.3389/fpls.2021.713890

**Published:** 2021-08-17

**Authors:** Tianheng Ren, Tao Fan, Shulin Chen, Xia Ou, Yongyan Chen, Qing Jiang, Yixin Diao, Zixin Sun, Wanhua Peng, Zhenglong Ren, Feiquan Tan, Zhi Li

**Affiliations:** ^1^College of Agronomy, Sichuan Agricultural University, Chengdu, China; ^2^Provincial Key Laboratory for Plant Genetics and Breeding, Chengdu, China

**Keywords:** wheat, QTL mapping, kernel size, wheat55K, KASP

## Abstract

As an important component, 1,000 kernel weight (TKW) plays a significant role in the formation of yield traits of wheat. Kernel size is significantly positively correlated to TKW. Although numerous loci for kernel size in wheat have been reported, our knowledge on loci for kernel area (KA) and kernel circumference (KC) remains limited. In the present study, a recombinant inbred lines (RIL) population containing 371 lines genotyped using the Wheat55K SNP array was used to map quantitative trait loci (QTLs) controlling the KA and KC in multiple environments. A total of 54 and 44 QTLs were mapped by using the biparental population or multienvironment trial module of the inclusive composite interval mapping method, respectively. Twenty-two QTLs were considered major QTLs. BLAST analysis showed that major and stable QTLs *QKc.sau-6A.1* (23.12–31.64 cM on 6A) for KC and *QKa.sau-6A.2* (66.00–66.57 cM on 6A) for KA were likely novel QTLs, which explained 22.25 and 20.34% of the phenotypic variation on average in the 3 year experiments, respectively. Two Kompetitive allele-specific PCR (KASP) markers, *KASP-AX-109894590* and *KASP-AX-109380327*, were developed and tightly linked to *QKc.sau-6A.1* and *QKa.sau-6A.2*, respectively, and the genetic effects of the different genotypes in the RIL population were successfully confirmed. Furthermore, in the interval where *QKa.sau-6A.2* was located on Chinese Spring and *T. Turgidum* ssp. *dicoccoides* reference genomes, only 11 genes were found. In addition, digenic epistatic QTLs also showed a significant influence on KC and KA. Altogether, the results revealed the genetic basis of KA and KC and will be useful for the marker-assisted selection of lines with different kernel sizes, laying the foundation for the fine mapping and cloning of the gene(s) underlying the stable QTLs detected in this study.

## Introduction

Common wheat (*Triticum aestivum* L.) is one of the most important food crops in the world and provides ~20% of the protein and calories in the human diet (Chaves et al., 2013). In recent years, due to the decrease in arable land area, population growth, and many other factors, it has been difficult for grain production to meet human demand. It is very important to increase the yield of common wheat to relieve the pressure on grain and meet social demand.

The 1,000 kernel weight (TKW) and kernel number per spike (KN) have important effects on the wheat yield, and they are usually considered key factors of yield formation. Previous studies showed that compared with the KN, the TKW might have a higher heritability, with a heritability range of 0.59–0.80 (Xiao and He, 2003). The kernel size is significantly positively correlated with the TKW. Therefore, in the long history of wheat breeding and domestication, the kernel size has been a major selection and breeding objective and has been widely used for selection to improve wheat yield (Gegas et al., 2010). Larger seeds usually showed a better yield and commerciality. The genetic improvement of kernel size is beneficial to increase the TKW and thus increase the yield of common wheat.

The kernel traits of wheat are complex agronomic traits and are controlled by multiple genes (Li et al., 2018). In previous studies, several quantitative trait loci (QTLs) or genes related to kernel traits have been identified and mapped, which were distributed on all of the chromosomes of common wheat (Gegas et al., 2010; Prashant et al., 2012; Tyagi et al., 2015; Kumar et al., 2016; Brinton et al., 2017; Yan et al., 2017; Li et al., 2018; Ma et al., 2019a; Cao et al., 2020; Liu et al., 2020). For instance, Tyagi et al. (2015) mapped QTLs related to kernel traits on 19 wheat chromosomes, except for the 2D and 3D chromosomes. Ma et al. (2019a) mapped a major QTL on a 2D chromosome that controls the kernel length (KL), kernel width (KW), and TKW. Liu et al. (2020) identified QTLs related to the TKW in the DH population and distributed them to wheat 1A, 2D, 4B, 4D, 5A, 5D, 6A, and 6D chromosomes. Several studies also indicated that a QTL across the dwarf gene *Rht-B1* on 4BS could affect both the KW and TKW (Ramya et al., 2010; Gao et al., 2015; Li et al., 2018; Ren et al., 2021). Although the cloning of related genes in common wheat is relatively slow due to its large genome (approximately 17.9 Gb) and high repeat sequence content compared with rice and maize (Simmonds et al., 2014; Su et al., 2018), to date, many genes related to the kernel traits of wheat have been cloned and verified. For instance, *TaGW2* on 6A was identified as a TKW QTL in a mapping population (Simmonds et al., 2014), and then the analysis of the haplotype structure of the 6A chromosome explains that the QTL region detected for KC and KA is different from the TKW QTL on 6A (Brinton et al., 2020). Moreover, *TaGW2* and *TaGS5* were also cloned based on their homologous genes *OsGW2* and *OsGS5* in rice, respectively (Wang et al., 2016; Zhai et al., 2018). *TaMOC1-7A* was cloned based on the *MOC1* gene in rice, and it was found that it could affect both the TKW and number of spikelets (Zhang et al., 2015). Several genes related to the TKW, such as *TaSnRK2.3-1A* (Miao et al., 2017)*, TaSnRK2.3-1B* (Miao et al., 2017)*, TaSUS2-2A* (Hou et al., 2014)*, TaCwi-A1* (Ma et al., 2012)*, TaFlo2-A1* (Sajjad et al., 2017)*, TaSUS2-2B* (Jiang et al., 2011), and *Tabas1-B1* (Zhu et al., 2016), were also cloned. More QTL mapping of kernel traits is ongoing, which laid a foundation for future gene cloning and application. Although numerous loci for the kernel traits in wheat have been reported, our knowledge on loci for the kernel area (KA) and kernel circumference (KC) remains limited (Kumari et al., 2018). Moreover, although several QTLs for the TKW, KL, and KW have been reported, most of them showed strong QTL × genotype and epistatic QTL × QTL interactions, which limited their further application in molecular marker-assisted breeding (MAS) (Campbell et al., 2003; Gupta et al., 2007; Prashant et al., 2012; Cabral et al., 2018).

Genetic linkage maps play an important role in the analysis of genetic components of agronomic traits. The large genetic distance between QTLs and flanking molecular markers often limits their application in MAS. With the development of high-throughput sequencing technology, high-density genetic linkage maps have been increasingly widely used in the study of various crops, such as rice (Xie et al., 2010), maize (Chen et al., 2014), eggplants (Barchi et al., 2012), and grapes (Wang et al., 2012). In recent years, high-density genetic linkage maps based on SNP micro-arrays have played an important role and have been widely used for QTL mapping for various agronomic traits in common wheat (Cui et al., 2017; Sun et al., 2020). Due to its economy and practicability, the Wheat55K SNP array has been employed for many gene mapping studies (Li et al., 2018; Ren et al., 2018; Huang et al., 2019; Ma et al., 2019b; Zhang et al., 2019).

In the present study, QTLs for KA and KC were identified using a genetic map constructed with the Wheat55K SNP array and phenotypic observations in multiple environments. Major QTL for KA and KC were further validated in the RIL population by KASP markers. The major and stable QTLs detected in this study will be useful for further MAS and map-based cloning.

## Materials and Methods

### Plant Materials

The elite wheat cultivar Chuannong18, which was released by the Sichuan Provincial Variety Examination and Committee in 2003, has been planted in southwestern China on nearly one million hectares. T1208 is a high-yield wheat line developed by our laboratory. In the present study, a 371 RIL population developed by the cross Chuannong18 (CN18) and T1208 was used for QTL analyses based on a high-density genetic linkage map constructed by the Wheat55K SNP array (Hu et al., 2017; Ren et al., 2018, 2021).

### Phenotypic Evaluation

The 371 lines of the RIL population and the two parents were planted in the Qionglai District, Chengdu Plain, Sichuan Province, China (30°250'N, 103°28'E, altitude 493.3 m) in the 2016, 2018, and 2019 cropping seasons. A randomized complete block design with three replications was used for the field experiment. Each plot was 3 m long and consisted of four rows with a 25 cm spacing between rows. The plant density was 160 seedlings per square meter. Cultivation management was the same as the field standardized management method in the Chengdu Plain. Herbicides and fungicides were applied to ensure that there were no pests or diseases in the field during the growth process and to avoid influencing the corresponding agronomic traits. One square meter from the central part of each plot was harvested to determine the kernel traits. Kernels were based on 12% moisture when measured (seeds were dried and measured by a grain moisture tester, PM-8188, Kett, Tokyo, Japan). The KA and KC were measured by a Wanshen SC-G automatic seed test analyzer (Hangzhou Wanshen Testing Technology Co., Ltd.). During the measurement, more than 100 seeds of each line were scanned for analysis. After scanning and taking pictures, each seed would be circled and the area of each seed and the circumference of each seed would be calculated by software. This method provides new parameters to determine the size of the kernels. And the TKW, KL, KW, kernel diameter ratio (KDR), and kernel weight per spike (KWPS) of each line were also calculated by the seed test analyzer, and the plant height (PH) was measured directly in the field during the filling stage. Those data were retrieved from our previous studies (Ren et al., 2021, Supplementary Table 1).

### Phenotypic Data Analyses

The average measurement of each trait of three replicates was used in the subsequent analysis. The heritability and ANOVA of each trait were performed as described previously (Hu et al., 2017; Ren et al., 2021). The Pearson's correlation coefficient was calculated using SPSS Ver. 22.0 (IBM SPSS, Armonk, NY, United States). For each trait, the best linear unbiased estimation (BLUE) was calculated across environments using the ANOVA function in IciMapping Ver4.1 assuming fixed effects for the genotype (Lin et al., 2020).

### QTL Mapping

A high-density genetic linkage map constructed previously using the Wheat55K SNP array was used in this study (Ren et al., 2021). In the RIL population, the markers were distributed in 21 linkage groups and covered a total genetic distance of 4192.62 cM with mean, minimum, and maximum marker densities of 0.36, 0.13, and 1.01 cM between adjacent markers, respectively (Ren et al., 2021). We used IciMapping 4.1 based on the biparental populations (BIPs) module with inclusive composite interval mapping (ICIM, http://www.isbreeding.net) (Meng et al., 2015) for QTL detection in the RIL population. The values of the KA and KC in each replicate of different years were assembled to conduct combined QTL analysis to identify the combined QTLs with additive-by-environment (A by E) interactive effects in an MET module using the ICIM method (Meng et al., 2015). The parameters of QTL analysis were set as follows: logarithm of odds (LOD) = 1,000 permutations, step = 1 cM, and PIN = 0.001. The CI of each QTL was determined by LOD > 3. An epistatic analysis was also performed by the IciMappingVer.4.1 EPI (epistatic) module, and the default parameter settings were used (LOD = 5, step = 1 cM, and stepwise regression probability < 0.0001) (Meng et al., 2015; Ren et al., 2021). QTLs were named based on the International Rules of Genetic Nomenclature (http://wheat.pw.usda.gov/ggpag es/wgc/98/Intro.htm). “Kc” represents the kernel circumference, “Ka” represents the kernel area, and “Sau” represents the Sichuan Agricultural University.

The flanking molecular markers of QTLs were compared with the Chinese Spring genome reference sequence [International Wheat Genome Sequencing Consortium (IWGSC) RefSeq version 1.0; https://urgi.versailles.inra.fr/download/iwgsc/] and *T. Turgidum* ssp. *dicoccoides* genome reference sequence (The *Triticeae* Multiomics Center, http://202.194.139.32/). After the physical locations of the QTL region were determined, the information of genes or sequences of the regions was downloaded from Wheatmine (https://urgi.versailles.inra.fr/WheatMine/begin.do). Finally, sequence alignment was performed, and the functions of the genes or sequences involved in these QTL regions were analyzed on the UniProt website (http://www.UniProt.org/).

### Marker Development and QTL Validation

By finding the sequence of the Wheat55K SNP molecular markers located in the QTL interval, the KASP primers were designed on the PolyMarker online website (http://www.polymarker.info/). A set of one KASP primer contained a total of three primer sequences: forward primer 1, forward primer 2, and a universal primer. The 5′ end of the forward primer 1 needs to add the FAM fluorophore group (5′-GAAGGTGACCAAGTTCATGCT- 3′), and the 5' end of the forward primer 2 needs to add the HEX fluorophore group (5′- GAAGGTCGGAGTCAACGGATT- 3′). All of the sequences of KASP primers designed in this experiment are listed in **Table 4**. A total of 127 lines for the KC and 128 lines for the KA were randomly selected from the RIL populations and used to perform genotyping using these KASP markers.

The PCRs were performed in a total volume of 10 μl containing 4.5 μl of 1×KASP Master mixture (JasonGen, www.jasongen.com), 2 μl (50 ng/μl) of template DNA, 2 μl of KASP primer mixture (forward primer 1:forward primer 2: universal primer: ddH_2_O = 6:6:15:23), and 1.5 μl of ddH_2_O. The whole process was carried out on real-time PCR (BioRad, CFX-96) system. The PCR procedure was as follows: 10 min at 95°C and 30 cycles of 20 s at 95°C and 40 s at 61–55°C (drop 0.6°C, per cycle).

## Results

### Phenotypic Analyses

**Figure 1:**
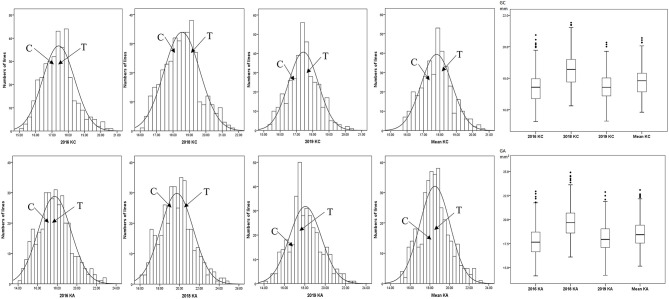
Phenotypic frequency distribution and box plot of kernel size in RIL Population. C, CN18; T, T1208.

The KC and KA values of CN18 were lower than those of T1208 in the phenotypic data of the 3 year experiments (Table 1, Figure 1, Supplementary Table 2). The KC values of CN18 and T1208 were in the ranges of 16.97–18.13 and 17.46–19.02 mm in the 3 years of data, respectively. The KA values of CN18 and T1208 were in the ranges of 17.55–19.00 mm^2^ and 17.71–20.64 mm^2^ in the 3 years of data, respectively. In the RIL population, the KC and KA showed continuous variation, and both had the phenomenon of transgressive inheritance (Table 1, Figure 1). The absolute value of skewness and kurtosis of the phenotypic distribution in the population was <1 (Table 1), which is consistent with a normal distribution and is a typical quantitative trait (Figure 1). Moreover, the KA and KC exhibited a high *h*^2^ (broad-sense heritability); the *h*^2^ of KA was 0.92, and the *h*^2^ of KC was 0.88. The coefficient of variation (CV) of KC was lower than that of KA, which suggested that KA had a higher degree of variation. A significant positive correlation was observed between the KC and KA in all the three environments (Table 2). It was also indicated that these two kernel-size traits are mainly affected by genetic factors, while environmental factors have less influence.

**Table 1:** Phenotypic variation and heritability of characters in different environments in parents and populations.

**Traits**	**Parental lines**		**Populations**						
	**CN18**	**T1208**	**Mean**	**Range**	**SD**	**CV%**	**Kurtosis**	**Skewness**	***h^**2**^***
KC 2016	17.16	17.46	17.41	15.24–20.75	0.99	5.68	0.486	0.344	
KC 2018	18.13	19.02	18.54	16.24–21.53	1.09	5.87	0.243	−0.270	
KC 2019	16.97	17.53	17.43	15.27–20.27	0.91	5.19	0.164	0.014	
KC Mean	17.42	18.00	17.80	15.84–20.56	0.95	5.45	0.242	−0.114	0.88
KA 2016	17.55	17.77	17.73	14.14–22.93	1.68	9.47	0.388	0.025	
KA 2018	19.00	20.64	19.72	16.10–24.90	1.66	8.41	0.339	0.017	
KA 2019	17.56	17.71	18.05	14.20–22.86	1.56	8.64	0.349	−0.047	
KA Mean	18.04	18.70	18.51	15.15–23.07	1.55	8.37	0.331	0.003	0.92

*CV, coefficient of variation; SD, standard deviation; h^2^, broad-sense heritability; KA, kernel area (mm^2^); KC, kernel circumference (mm)*.

**Table 2:** Correlation coefficients of kernels-related traits in different environments.

	**2016 KC**	**2018 KC**	**2019 KC**	**2016 KA**	**2018 KA**	**2019 KA**	**Mean KA**
2016 KC	1						
2018 KC	0.853^**^	1					
2019 KC	0.796^**^	0.886^**^	1				
Mean KC	0.932^**^	0.968^**^	0.939^**^	0.976^**^	0.974^**^	0.890^**^	0.952^**^
2016 KA				1			
2018 KA				0.993^**^	1		
2019 KA				0.774^**^	0.771^**^	1	

*KC, kernel circumference; KA, kernel area*;

** *significance level at P < 0.01*.

### QTL Analysis in Individual Environments

Using the ICIM–BIP method, 54 additive QTLs for the KC and KA were detected, and these QTLs were mapped to chromosomes 1A, 2A, 2B, 2D, 3D, 4A, 4B, 4D, 5A, 5B, 5D, 6A, 6B, 6D, 7A, 7B, and 7D (Supplementary Table 3). Among them, 22 QTLs that were detected during more than 1 year (or in the mean data) or explained >10% of the phenotypic variation and had an LOD > 3 were considered major QTLs and are listed in Table 3.

**Table 3:** Major QTL mapping results of single environment analysis.

**Traits**	**QTL**	**Year**	**Interval (cM)**	**Closet marker**	**LOD**	**PVE (%)**	**Add**
KC	QKc.sau-2A	Y18/Mean	147.62–148.33	AX-111014053	10.09	5.01	0.18
	QKc.sau-2D.2	Y19/Mean	297.61–299.08	AX-86176576	10.71	4.60	0.17
	QKc.sau-3D.1	Y16/Mean	54.70–58.40	AX-108923200	22.31	11.65	0.26
	QKc.sau-4A.2	Y18/Mean	107.88–108.02	AX-109918502	9.71	4.00	−0.16
	QKc.sau-4B.2	Mean	75.93–77.06	AX-111571347	52.84	21.56	−0.46
	QKc.sau-5A.1	Y16/Y18/Mean	56.75–80.36	AX-108871400	9.03	6.18	−0.20
	QKc.sau-5B.1	Y16	150.91–151.20	AX-109321350	17.01	10.25	−0.25
	QKc.sau-5B.2	Y18/Y19	162.79–163.07	AX-110558483	9.35	5.40	−0.18
	**QKc.sau-6A.1**	Y16/Y18/Y19/Mean	23.12–31.64	AX-109894590	33.11	22.25	0.42
	QKc.sau-6A.2	Y19	33.74–46.70	AX-110928314	18.51	12.07	−0.28
	QKc.sau-7D.2	Y16	111.97–112.11	AX-109583841	16.84	10.05	0.25
KA	QKa.sau-1A	Y16/Mean	54.64–55.20	AX-111149806	4.63	2.01	−0.20
	QKa.sau-2A.1	Y18/Mean	147.62–148.33	AX-111014053	5.71	2.26	0.21
	QKa.sau-3D	Y16/Y18	170.87–173.58	AX-95008504	9.93	4.03	0.31
	QKa.sau-4B	Y16/Y18/Mean	67.53–72.39	AX-110472645	8.08	3.36	−0.27
	QKa.sau-4D.1	Y18/Y19/Mean	94.86–123.09	AX-108786137	5.49	3.63	0.26
	QKa.sau-5B	Y18/Y19/Mean	150.91–151.20	AX-109321350	9.99	4.44	−0.30
	QKa.sau-5D.1	Y16/Y18/Mean	91.93–101.11	AX-95229410	8.61	4.05	−0.30
	QKa.sau-6A.1	Y16/Y18/Mean	23.12–31.64	AX-109894590	26.33	13.51	0.58
	**QKa.sau-6A.2**	Y16/Y18/Y19/Mean	66.00–66.57	AX-108852271	39.56	20.34	−0.63
	QKa.sau-6A.3	Y19	66.85–79.35	AX-110040743	24.44	15.16	0.63
	QKa.sau-6D.3	Y16/Y18/Mean	218.76–219.04	AX-111466043	30.56	14.45	0.58

*LOD, likelihood of odds; PVE (%), proportion of phenotypic variation of the corresponding QTL; ADD, additive effect*.

*Positive additive effects indicate that alleles from T1208 enhance corresponding trait value, and negative additive effects indicate that alleles from CN18 enhance the corresponding trait value. The bold font showed two stable and major QTLs mapped in this study*.

**Figure 2:**
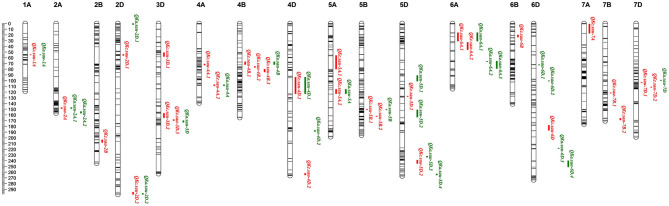
The additive QTLs for kernel area (KA) and kernel circumference (KC) in the genetic map. Red for KC and green for KA. Thirty KC and 24 KA QTLs were mapped to chromosomes 1A, 2A, 2B, 2D, 3D, 4A, 4B, 4D, 5A, 5B, 5D, 6A, 6B, 6D, 7A, 7B, and 7D, respectively. The major and stable QTLs *QKc.sau.6A.1* (23.12–31.64 cM, closet marker: AX-109894590) and *QKa.sau.6A.2* (66.00–66.57 cM, closet marker: AX-108852271) were mapped on 6A chromosome. The positions, interval, and closet markers of other QTLs are listed in Table 3. The scale label of the linkage map is 2 cM, which is showed in the left.

Thirty KC QTLs were mapped (Table 3, Supplementary Table 3, and Figure 2). The LOD of each QTL ranged from 4.22 to 42.65, and each QTL explained 1.28–34.68% of the KC variation. The additive effect values of 16 QTLs were negative, indicating that the genetic effects of these QTLs were derived from CN18. The other 14 QTLs were derived from T1208 because their additive effect values were positive (Supplementary Table 3). Among them, 11 QTLs were considered as major QTLs. *QKc.sau.6A.1* was mapped in the interval of the 6A chromosome (23.12-31.64 cM) in 3 years and explained 9.77, 29.73, and 34.68% of the phenotypic variation, respectively (Table 3). The additive effect values of *QKc.sau.6A.1* were positive, indicating that the genetic effects of *QKc.sau.6A.1* were derived from T1208. Moreover, *QKc.sau-5A.1* (56.75–80.36 cM on 5A) and *QKc.sau-5B.2* (162.79–163.07 cM on 5B) were mapped in 2 years and explained 2.8–8.31% and 4.33–6.47% of the phenotypic variation, respectively (Supplementary Table 3, Table 3).

Twenty-four KA QTLs were also mapped (Table 3, Supplementary Table 3, and Figure 2). The LOD of each QTL ranged from 3.28 to 47.61, and each QTL explained 1.29–23.40% of the KA variation. Eleven QTLs and 13 QTLs were derived from CN18 or T1208, respectively, due to their additive effect values (Supplementary Table 3). Among them, 11 QTLs were considered major QTLs. *QKa.sau-6A.2* was mapped in the interval of the 6A chromosome (66.00–66.57 cM) in all the 3 years and explained 20.99, 21.86, and 15.12% of the phenotypic variation, respectively (Table 3). The additive effect values of *QKa.sau.6A.2* were negative, indicating that the genetic effects of *QKa.sau.6A.2* were derived from CN18. In addition, *QKa.sau-4B* (67.53–72.39 cM on 4B), *QKa.sau-4D.1* (94.86–123.09 cM on 4B), *QKa.sau-5B* (150.91–151.20 cM on 5B), *QKa.sau-5D.1* (91.93–101.11 cM on 5D), *QKa.sau-6A.1* (16.78–34.64 cM on 6A), and *QKa.sau-6D.3* (218.76–219.04 cM on 6A) were mapped in 2 years and explained 2.17–4.44%, 1.29–5.24%, 3.44–5.44%, 2.34–5.57%, 11.98–16.22%, and 13.97–15.08% of the phenotypic variation, respectively (Supplementary Table 3, Table 3).

### Combined QTL-by-Environment Interaction Analysis

Using the MET method of ICIM, 24 cQTLs of the KC, and 20 cQTLs of the KA were mapped (Supplementary Table 4).

In the MET analysis, 20 cQTLs of the KC and 17 cQTLs of the KA were the same as the QTL analysis by the BIP method. The major QTLs that could be mapped for more than 2 years (three for KC and eight for KA) by the ICIM–BIP method were also mapped by the MET method (Supplementary Table 4, Table 3). Among these cQTLs for the KC and KA, PVE (A) (representing phenotypic variation explained by additive and dominance effects) ranged from 0.26 to 37.33% and 0.24 to 29.20%, respectively. The PVE (A by E) (additive and dominance by environment effects for corresponding QTLs) were in the ranges of 0.26–4.44% and 0.09–5.22%, respectively. The value of PVE (A by E) was significantly lower than the value of PVE (A). This indicates that both the KA and KC are mainly influenced by genetic factors.

The results also showed that the phenotypic variation of KC explained by the interactive effects of the environment and *cQKc.sau-6A.1* was small [PVE (A by E) = 1.00%], while the phenotypic variation explained by additive and dominance effects was high [PVE(A) = 37.33%]. This indicates that *QKc.sau-6A.1* is a stable and major QTL for KC. On the other hand, the PVE (A by E) of *cQKa.sau-6A.2* was only 0.13%, and the PVE (A) was 29.20%. This indicates that *QKa.sau-6A.2* is a stable and major QTL for KA.

### Epistatic Analysis

**Figure 3:**
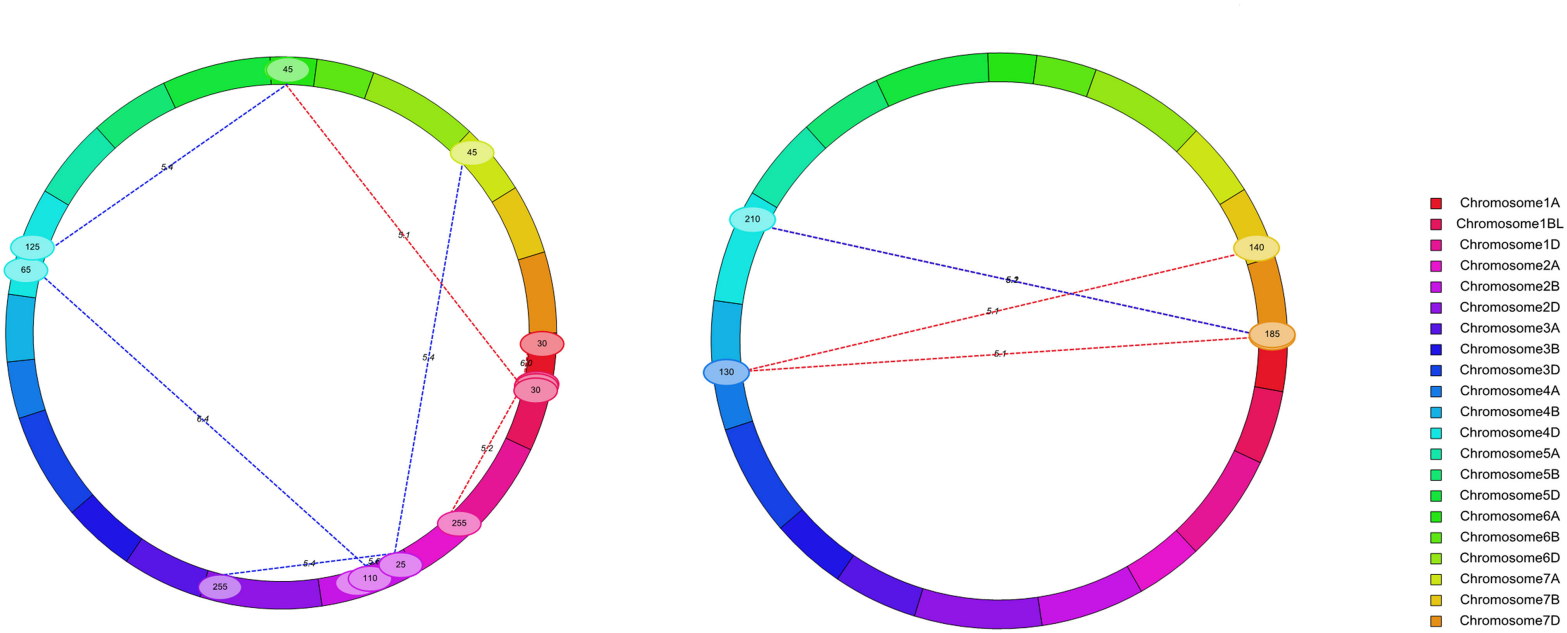
Chromosomal locations of digenic epistatic QTL for KC (left) and KA (right). A total of nine digenic epistatic QTLs of KC and four digenic epistatic QTLs of KA were mapped, respectively.

A total of nine digenic epistatic QTLs of KC and four digenic epistatic QTLs of KA were mapped (Supplementary Table 5, Figure 3) and explained 8.30–21.28% and 5.25–14.36% of the phenotypic variation, respectively (Supplementary Table 5).

The negative epistatic effect values (add by add) suggest that the epistatic effect of the recombinant genotype was higher than that of the parental genotype. The epistatic effect values (add by add) of four KC QTLs were negative, and five of them were positive (Supplementary Table 5). This result suggested that both the recombinant genotype and the parental genotype have epistatic effects on the KC. In contrast, the epistatic effect values (add by add) of all the four QTLs for KA were positive. This result suggested that the epistatic effect of the recombinant genotype was smaller than that of the parental genotype on the KA (Supplementary Table 5).

Among these epistatic QTLs, *eQKc.sau-6A* corresponded to *QKc.sau-6A.2*, which was mapped by the BIP method. Digenetic epistatic additive effects were found between *eQKc.sau-6A* and two loci (*eQKc.sau-1BL* and *eQKc.sau-4D.2*) and explained 8.52 and 8.91% of the phenotypic variation, respectively (Supplementary Table 5). It is suggested that the KC is influenced by both additive and dominant effects and epistatic effects. On the other hand, the KA is mainly affected by the interaction between random loci. These random loci indirectly influence the phenotype through interactions with each other. Moreover, digenetic epistatic additive effects were found between *eQKc.sau-4D.1* and *eQKc.sau-2B.2* for the KC, which explained 21.28% of the phenotypic variation but were only detected in 1 year. And digenetic epistatic additive effects were found between *eQKa.sau-7D.1* and *eQKa.sau-4D* for the KA, which explained 14.36% of the phenotypic variation and were detected in 2016 and 2018 (Supplementary Table 5). It is suggested that although epistasis plays a large role in phenotypes, it is not stable.

### KASP Marker Development and Validation of Two Stable QTLs

**Figure 4:**
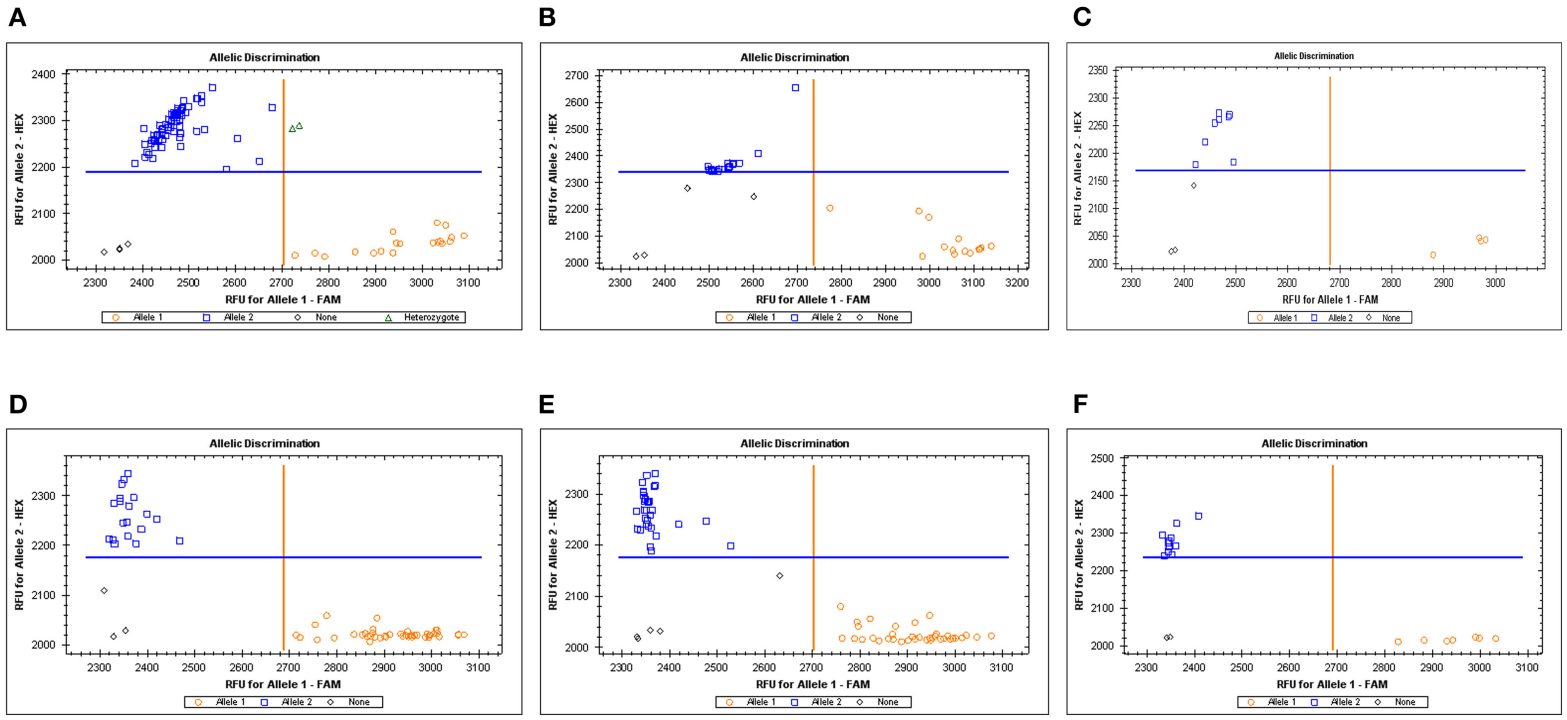
Genotyping results of KASP markers related to KC in RIL population **(A–C)** and KA in RIL population **(D–F)**. *KASP-AX-109894590* (For *QKc.sau-6A.1*) and *KASP-AX-109894590* (*QKa.sau-6A.2*) were used to identify the alleles in the RIL population and were classified into two groups.

**Figure 5:**
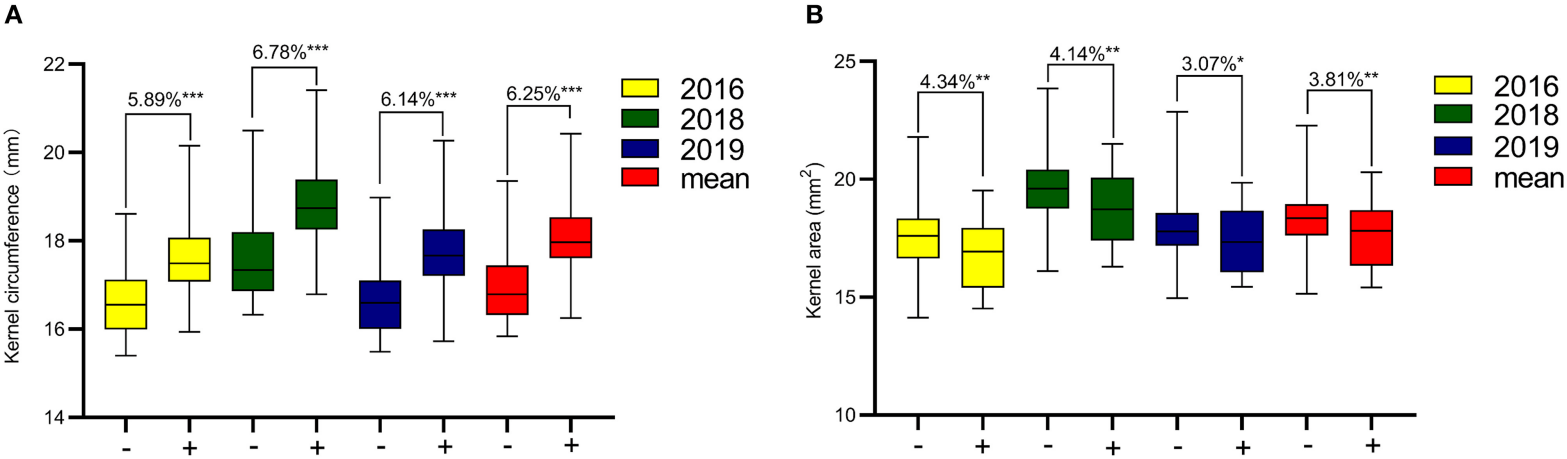
Effects of *QKc.sau-6A.1***(A)** and *QKa.sau-6A.2* **(B)** in RIL population. “+” Indicates the lines with the genotypes “*AA*” or “*BB*,” “–” indicates the lines without the genotypes “*aa*” or “*bb*.” *, **, and ***, significant at *P* < 0.05, 0.01, and 0.001, respectively.

Based on the QTL mapping results and sequences of the molecular markers of SNP array, two KASP markers were developed and used to validate two stable QTLs. The KASP marker *KASP****-****AX-109894590* was closely linked to *QKc.sau-6A.1*, and *KASP-AX-109380327* was closely linked to *QKa.sau-6A.2* (Table 4). These KASP markers were used to identify the genotype. For *QKc.sau-6A.1, KASP-AX-109894590* was used to identify the alleles in the RIL population and were classified into two groups. A total of 127 lines were randomly selected from the RIL population for genotyping using *KASP-AX-109894590* molecular markers. Among the 127 lines, 33 lines had the same genotype as CN18, while 94 lines had the same genotype as T1208 (Figure 5, Figures 4A–C). The genotype “*AA*” shows that *QKc.sau-6A.1* carried the homozygous alleles from T1208. And the genotype “*aa*” shows that *QKc.sau-6A.1* carried the homozygous alleles from non-T1208. The results of the *t*-test showed that the “*AA*” genotypes had higher phenotypic values of KC in all environments and in the mean values than the “*aa*” genotypes (*P* < 0.001) (Figure 5A). For *QKa.sau-6A.2, KASP-AX-109380327* was used to identify the alleles in the RIL population and were also classified into two groups. A total of 128 lines were randomly selected from the RIL population for genotyping using *KASP-AX-109380327* molecular markers. Among the 128 lines, 49 lines had the same genotype as CN18, while 79 lines had the same genotype as T1208 (Figure 5, Figures 4D–F). The genotype “*BB*” shows that *QKa.sau-6A.2* carried the homozygous alleles from CN18. And the genotype “*bb*,” shows that *QKa.sau-6A.2* carried the homozygous alleles from non-CN18. The results of the *t*-test showed that the “*BB*” genotypes had lower phenotypic values of KA in all environments and in the mean values than the “*bb*” genotypes (*P* < 0.001) (Figure 5B).

**Table 4:** Kompetitive allele-specific PCR markers for *QKc.sau-6A.1* and *QKa.sau-6A.2*.

**QTL**	***QKc.sau-6A.1***	***QKa.sau-6A.2***
Marker	*KASP-AX-109894590*	*KASP-AX-109380327*
Forward primer 1 (5'to3')	CCTTATCTTGGCCAGT TCATAAC	GAGTAGCCTCCTACC CATTATTG
Forward primer 2 (5'to3')	CCTTATCTTGGCCAGT TCGTAAT	GAGTAGCCTCCTACCC ATTATTC
Reverse primer (5'to3')	TGACAGCCAAGGG AACACAT	TTACTGCCCATT CACGCTGA

*KASP, Kompetitive allele-specific PCR*.

*The probe sequence is not included in the above primer sequence. FAM probe sequence of the forward primer is GAAGGTGACCAAGTTCATGCT, and HEX probe sequence of the reverse primer is GAAGGTCGGAGTCAACGGATT*.

### Effects of the Two Major QTLs Related to Other Yield Traits

**Figure 6:**
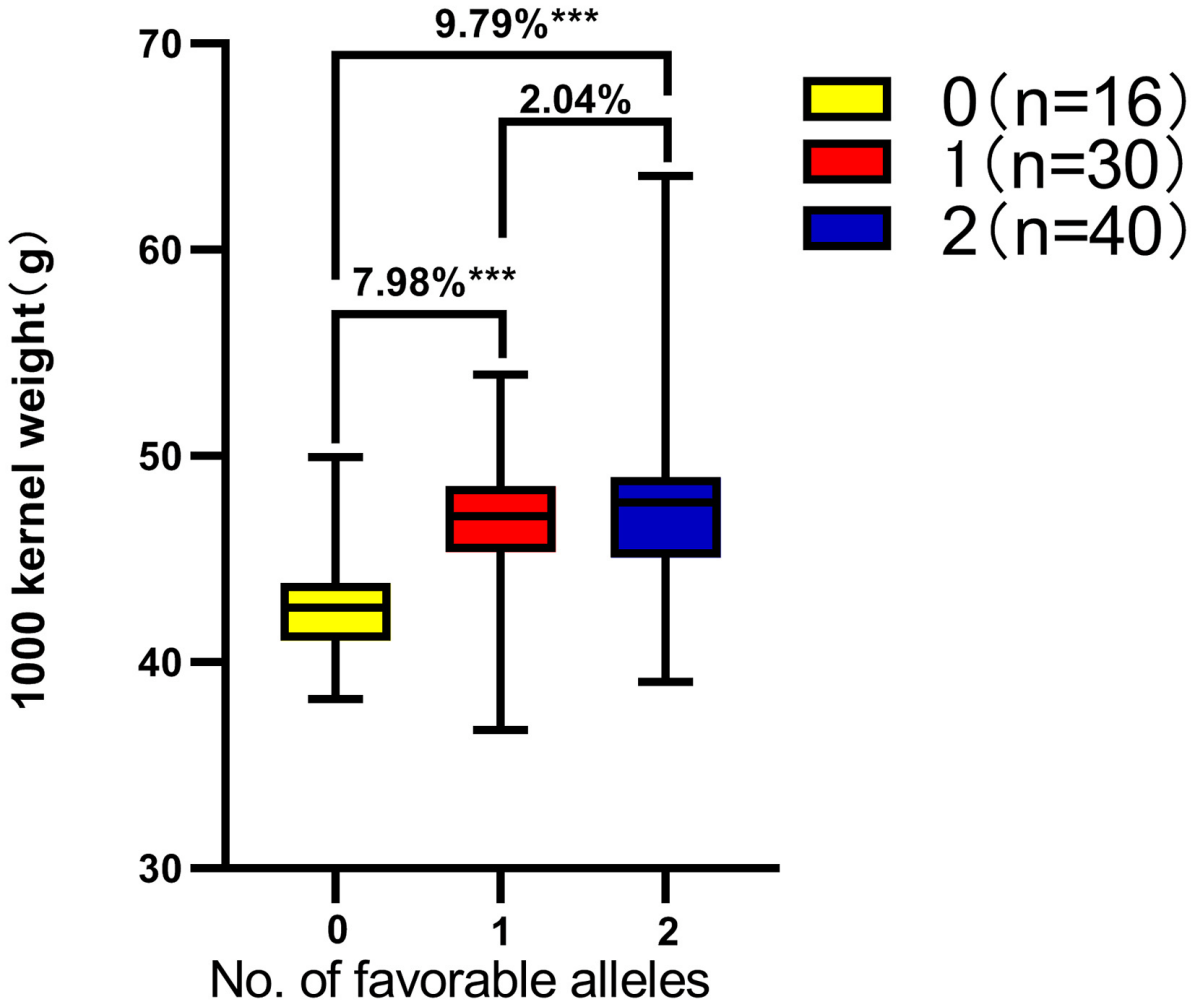
Number of favorable alleles for two kernel-related QTLs on thousand grain weight in the Chuannong18/T1208 RIL population based on the across-environment BLUEs. ***Significant at *P* < 0.001. Genotype of 40 lines were *AAbb*, 30 lines were *AABB* or *aabb*, and 16 lines were *aaBB*. The TKW of the *AAbb, AABB*, and *aabb* genotype lines was significantly higher than that of the *aaBB* genotype lines.

Based on the genotyping results of *KASP-AX-109894590* and *KASP-AX-109380327* molecular markers and the BLUE value of each yield trait, the effects of *QKc.sau-6A.1* and *QKa.sau-6A.2* on the other six yield traits were analyzed. The results showed that “*AA*” genotypes had significant positive effects on the PH, TKW, KL, KDR, and KWPS (*P* < 0.05) and had no significant effects on the KW (Table 5). On the other hand, “*BB*” genotypes had no significant effects on the PH and KW and had significant negative effects on the TKW, KL, KDR, and KWPS (*P* < 0.05) (Table 5). To further analyze the relationship between the TKW and these two QTLs, a total of 86 lines were randomly selected from the RIL population for analysis. Among these 86 lines, 40 lines were “*AAbb*” genotypes, 30 lines were “*AABB*” or “*aabb*” genotypes, and 16 lines were “*aaBB*” genotypes. The TKW of the “*AABB*” *or* “*aabb*” genotype lines was significantly higher than that of the “*aaBB*” genotype lines (7.98%). The TKW of the “*AAbb*” genotype lines were significantly higher than those of the “*aaBB*” genotype lines (9.79%). However, there were no significant differences between the “*AAbb*” genotype lines and the “*AABB*” or “*aabb*” genotype lines (Figure 6). The QTL *QKc.sau-6A.1* showed better effects on the TKW than the QTL *QKa.sau-6A.2*.

**Table 5:** Effects of *QKc.sau-6A.1* and *QKa.sau-6A.2* on yield-related traits according to BLUE value.

**QTL**	**Alleles**	**PH (cm)**	**TKW (g)**	**KL (mm)**	**KW (mm)**	**KWPS (g)**
*QKc.sau-6A.1*	*aa*	82.58	44.69	6.68	3.45	2.18
	*AA*	87.26^*^	49.32^***^	7.29^***^	3.49	2.53^***^
*QKa.sau-6A.2*	*BB*	81.77	45.20^**^	6.85^**^	3.46	2.23^***^
	*bb*	84.10	47.59	7.10	3.44	2.54

*BLUE, phenotype values based on best liner unbiased estimation*.

*** *Significant at p < 0.001*;

** *Significant at p < 0.01*;

* *significant at p < 0.05*.

### Genetic Analysis of QTL *QKa.sau-6A.2*

**Figure 7:**
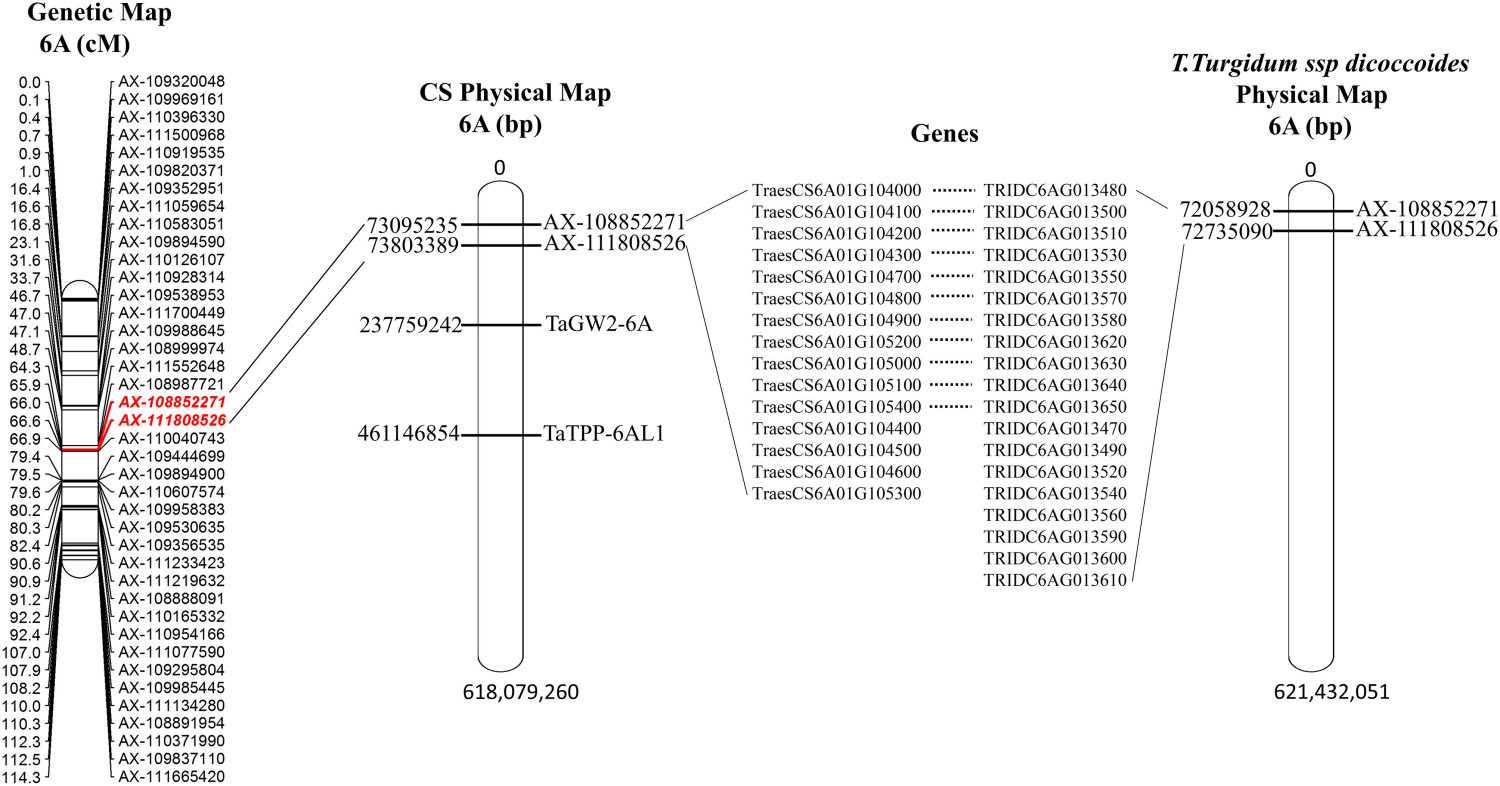
The physical position for *QKa.sau-6A.2* and its comparison with previous reported QTLs for kernel size. The two flanking molecular markers of *QKa.sau-6A.2* (*AX-108852271* and *AX111808526*) were located at 73.09–73.80 Mb of the physical map of the Chinese Spring genome reference sequence (73095235, 73803389) and 72.06–72.74 Mb of the physical map of the *T. Turgidum* ssp. *dicoccoides* genome reference sequence (72058928, 72735090). A total of 15 or 19 genes were included in this region in Chinese Spring and *T. Turgidum* ssp. *dicoccoides*, respectively.

The major and stable QTL *QKa.sau-6A.2*, which was related to the KA, was mapped on the 6A chromosome from 66.00 to 66.57 cM. The flanking molecular markers of *QKa.sau-6A.2* were compared with the Chinese Spring and *T. Turgidum* ssp. *dicoccoides* genome reference sequences. The interval region of this QTL was only 0.57 cM. The two flanking molecular markers of *QKa.sau-6A.2* (*AX-108852271* and *AX111808526*) were located at 73.09–73.80 Mb of the physical map of the Chinese Spring genome reference sequence (73095235, 73803389) and 72.06–72.74 Mb of the physical map of the *T. Turgidum* ssp. *dicoccoides* genome reference sequence (72058928, 72735090) (Figure 7). A total of 15 or 19 genes were included in this region in Chinese Spring and *T. Turgidum* ssp. *dicoccoides*, respectively, and 11 of them were the same (Supplementary Table 6, Figure 7).

## Discussion

### QTL Mapping of the KA and KC

The yield of wheat is a very complex agronomic trait that is a quantitative trait and controlled by multiple genes (Heidari et al., 2011). Among the yield-related traits of wheat, such as the flowering time, spike numbers per unit area, and number of kernels per spike, these traits tend to have lower heritability than the TKW and are more affected by environmental factors than the TKW (Xiao and He, 2003; Liu et al., 2020). The TKW is positively correlated with the wheat yield and is significantly affected by the kernel size. Kernel size-related traits are also controlled by multiple genes (Li et al., 2018; Yan et al., 2019; Yu et al., 2019). However, many studies on kernel size–related traits usually focused on the KL or KW (Bednarek et al., 2012; Mohler et al., 2016; Zhang et al., 2017; Ma et al., 2019a; Yang et al., 2019; Ren et al., 2021). In this study, to identify more QTLs or genes related to kernel size, we used scanning instruments combined with software analysis to carry out QTL mapping for KA and KC. The traditional methods for measuring the kernel size focused more on the KL and KW, and they revealed that KL and KW had a significant correlation with the TKW. However, in this study, compared with the KL and KW, the KC and KA showed a better coefficient of correlation with the TKW (Table 6). It is suggested that the KA or KC might be better parameters to reflect kernel size. However, KC/KA and TKW were controlled by different QTLs/genes in the wheat genome. For instance, Brinton et al. (2020) indicated that the QTLs for KC and KA were different from the TKW-controlling gene *TaGW2*. In this study, we mapped the two most stable and major QTLs for the KC and KA, namely, *QKc.sau-6A.1* and *QKa.sau-6A.2*, which were detected in multiple environments. The average LOD values of these two QTLs were as high as 30.11 and 39.56, while they could explain 22.25 and 20.34% of the phenotypic variation, respectively (Table 3). These two QTLs for KA and KC were also located on the different regions of the QTLs for TKW (Ren et al., 2021). It is indicated that, *QKc.sau-6A.1* and *QKa.sau-6A.2* were different from the QTLs for TKW.

**Table 6:** The coefficient of correlation of TKW with KL, KW, KC, and KA.

	**KL**	**KW**	**KC**	**KA**
TKW	0.808^**^	0.768^**^	0.847^**^	0.916^**^

** *Significance at the 0.01 probability level; KL, kernel length; KW, kernel width; KC, kernel circumference; KA, kernel area; TKW, 1,000 kernel weight*.

Previous studies have mapped several QTLs also related to the KA and KC (Xiao et al., 2011; Tyagi et al., 2015; Zhao et al., 2015; Yan et al., 2017; Kumari et al., 2018). However, we did not find the same QTL loci as *QKc.sau-6A.1* and *QKa.sau-6A.2* by comparing the physical locations of the molecular markers flanking both of them on the Chinese Spring reference genome. In addition, we compared the genes controlling kernel size on the 6A chromosome, such as *TaGW2-6A* (Bednarek et al., 2012) and *TaTPP-6AL1* (Zhang et al., 2017), but their physical locations were different from the two QTLs mapped in this study. It is suggested that *QKc.sau-6A.1* and *QKa.sau-6A.2* were two new stable and major QTLs. In addition, *QKc.sau-2A, QKc.sau-2B*, and *QKa.sau-2A.1* were consistent with the QTLs reported by Xin et al. (2020), which explained 5.01, 3.38, and 2.26% of the variation in this study, respectively. *QKc.sau-7B.2* and *QKa.sau-6D.3* were consistent with the QTLs reported by Xiao et al. (2011), which explained 4.22 and 14.45% of the variation in this study, respectively.

Previous studies showed that epistasis plays an important role in the inheritance of complex quantitative traits (Cao et al., 2001; Liao et al., 2001; Luo et al., 2001). In this study, the epistatic effects of the KC and KA were analyzed by using the ICIM method, and nine pairs and four pairs of digenic epistatic QTLs were detected, respectively. The results of epistatic analysis showed that there were interactions between one locus and several other loci in the detected epistatic QTLs. For instance, *eQKc.sau-2B.1* had epistatic effects with *eQKc.sau-2B.3* and *eQKc.sau-2D*, and *eQKa.sau-4A* had epistatic effects with *eQKa.sau-7B* and *eQKa.sau-7D.2* (Figure 3, Supplementary Table 5). However, their explained phenotypic variation values were different, indicating that the same locus had different epistatic effects on other loci. In addition, there were interactions between additive QTLs and random loci (Supplementary Table 5). It is also suggested that additive QTL not only directly affects phenotypic traits but also interacts with other loci to indirectly affect phenotypic traits. Several epistasis QTLs, such as *eQKc.sau-4D.1* and *eQKa.sau-7D.1*, showed high PVE for the phenotypic variation (Supplementary Table 5). The PVE of six pairs of digenic epistasis QTLs for KC was higher than 10%, and the other three were also close to 10%. It is suggested that KC is more susceptible to epistasis. However, most epistatic QTLs were only detected in 1 year, only few epistatic QTLs, such as *eQKa.sau-4D* and *eQKa.sau-7D.1* were detected more than 1 year (Supplementary Table 5). It is suggested that in addition to single additive QTLs, some epistatic QTLs also played important roles for KC, although they were not stable in every year.

### Molecular Marker Development and QTL Validation

Compared with traditional techniques, MAS is more accurate and more efficient, and it can usually greatly shorten the breeding period and reduce costs (Kuchel et al., 2007; Gupta et al., 2010). Therefore, in recent years, more attention has been given to the combination of MAS and traditional breeding technology, which may become a new breakthrough in crop breeding programs (Kuchel et al., 2007; Gupta et al., 2010). The rapid development of genome sequence techniques and QTL mapping methods has provided a powerful tool for the study of complex quantitative traits (Sun et al., 2020). A large number of QTLs and genes that affect yield, agronomy, quality, and biological and abiotic resistance have been identified by means of genome-wide linkage maps of molecular markers (Ramya et al., 2010; Simmonds et al., 2014; Gao et al., 2015; Yan et al., 2017; Li et al., 2018; Su et al., 2018; Ma et al., 2019a; Cao et al., 2020; Liu et al., 2020; Ren et al., 2021). KASP technology plays an important role in QTL mapping in wheat (Tan et al., 2017). The principle KASP is based on the reading of terminal fluorescence signals for judgment. Each reaction uses dual-color fluorescence to detect two genotypes of SNP sites, and different SNPs correspond to different fluorescent signals. KASP technology does not require the synthesis of specific fluorescent primers for each SNP site. Based on the unique ARM PCR principle, all SNP sites can be detected by universal fluorescent primer amplification (Semagn et al., 2014). KASP technology has the advantage of low cost and high accuracy, and thus, it has good application potential in wheat research (Allen et al., 2013; Hu et al., 2019).

In this study, two KASP markers, *KASP-AX-109894590* and *KASP-AX-109380327*, were developed and closely linked to *QKc.sau-6A.1* and *QKa.sau-6A.2*, respectively (Table 4). These two KASP markers were used to genotype more than 100 lines randomly selected from the RIL population. These two markers can genotype different alleles in the population (Figure 4). All of the lines having the same fluorescent signal as T1208 when tested by *KASP-AX-109894590* indicated that these lines carried the homozygous alleles “*AA*” from T1208, and these lines exhibited significantly higher KC and TKW (Figures 5, 6, Table 5). On the other hand, all of the lines having the same fluorescent signal as CN18 when tested by *KASP-AX-109380327* indicated that these lines carried the homozygous alleles “*BB*” from CN18, and these lines exhibited significantly lower KA and TKW (Figures 5, 6, Table 5). These results indicated that these two QTLs located on the 6A chromosome were validated. The major QTLs *QKc.sau-6A.1* and *QKa.sau-6A.2* that were mapped in this study might have an important influence on the heredity of kernel-related traits, and the KASP markers closely linked to them could be used for MAS in future breeding programs.

### Genetic Analysis for *QKa.sau-6A.2*

Fifteen candidate genes were mapped in the region from 73.09 to 73.80 Mb on chromosome 6AS of wheat, and 19 genes were mapped in the region from 72.06 to 72.74 Mb on chromosome 6AS of wild emmer wheat (Figure 7, Supplementary Table 6). Among them, 11 genes were the same. Some of those genes may be related to kernel traits. For instance, *TraesCS6A01G104300*, which encodes GDSL-like lipase, plays an important role in seed development and plant growth (Tan et al., 2014). The protein encoded by *TraesCS6A01G104200* has the domain of an F-box protein, which is involved in the nutrition as well as reproductive growth and development in many plants, and it can be used as the site of protein interaction to provide the basis for grain filling (Van den Burg et al., 2008; Ma et al., 2019a). However, the target genes need to be verified and confirmed by further studies.

## Data Availability Statement

The original contributions presented in the study are included in the article/Supplementary Material, further inquiries can be directed to the corresponding author/s.

## Author Contributions

TR and ZL designed the experiments. TR and ZR created the RIL population. TR, TF, SC, XO, YC, QJ, YD, ZS, WP, and ZR participated in phenotype measurement. TR, ZL, TF, and ZR did the field experiments. TR, ZL, and TF participated in data analysis and processing. TF performed QTL analysis. TR wrote the manuscript. All authors participated in the research and approved the final manuscript.

## Conflict of Interest

The authors declare that the research was conducted in the absence of any commercial or financial relationships that could be construed as a potential conflict of interest.

## Publisher's Note

All claims expressed in this article are solely those of the authors and do not necessarily represent those of their affiliated organizations, or those of the publisher, the editors and the reviewers. Any product that may be evaluated in this article, or claim that may be made by its manufacturer, is not guaranteed or endorsed by the publisher.
