# Supplementary material for: QTL Mapping and Validation for Kernel Area and Circumference in Common Wheat via High-Density SNP-Based Genotyping
Source: Front Plant Sci. 2021 Aug 17;12:713890. doi: 10.3389/fpls.2021.713890 (PMC8415916; doi:10.3389/fpls.2021.713890)
Supplement: Supplementary file 1 [file Table_1.DOCX]

**Table S1** Phenotypic variation and heritability of kernel-related traits in different environments in parents and RILs

| **Traits** | **Parental Lines** | |  | **RIL Populations** | | | | |
| --- | --- | --- | --- | --- | --- | --- | --- | --- |
|  | **CN18** | **T1208** |  | **Mean** | **Min-max** | **SD** | **CV%** | **h^2^** |
| **TKW** | 47.26 | 46.69 |  | 46.53 | 35.4-63.2 | 4.26 | 9.1 | 0.64 |
| **KL** | 6.88 | 7.30 |  | 7.10 | 6.06-8.29 | 0.44 | 6.2 | 0.65 |
| **KW** | 3.58 | 3.4 |  | 3.49 | 3.21-3.76 | 0.11 | 3.12 | 0.67 |
| **PH** | 90.1 | 76.8 |  | 84.1 | 49-114 | 12.79 | 15 | 0.92 |
| **KDR** | 1.92 | 2.15 |  | 2.04 | 1.73-2.39 | 0.127 | 6.2 | 0.89 |
| **KWPS** | 2.10 | 2.45 |  | 2.16 | 1.35-3.15 | 0.34 | 1.6 | 0.52 |

SD: standard deviation; CV :coefcient of variation; h^2^ broad-sense heritability; TKW:1000 kernel weight (g); KL: kernel length (mm); KW: kernel width (mm); KDR: kernel diameter ratio (KL/KW); KWPS: kernel weight per spike (g); PH：plant height (cm). The data were the mean values of three years’ data.
